# Mayaro Fever Virus, Brazilian Amazon

**DOI:** 10.3201/eid1511.090461

**Published:** 2009-11

**Authors:** Raimunda S.S. Azevedo, Eliana V.P. Silva, Valéria L. Carvalho, Sueli G. Rodrigues, Joaquim P. Nunes Neto, Hamilton A.O. Monteiro, Victor S. Peixoto, Jannifer O. Chiang, Márcio R.T. Nunes, Pedro F.C. Vasconcelos

**Affiliations:** Instituto Evandro Chagas, Ananindeua, Brazil (R.S.S. Azevedo, E.V.P. Silva, V.L. Carvalho, S.G. Rodrigues, J.P. Nunes Neto, H.A.O. Monteiro, J.O. Chiang, M.R.T. Nunes, P.F.C. Vasconcelos); Universidade do Estado do Pará, Belém, Brazil (V.S. Peixoto, P.F.C. Vasconcelos)

**Keywords:** Mayaro virus, outbreak, rash, febrile illness, genetic characterization, viruses, Brazilian Amazon, dispatch

## Abstract

In February 2008, a Mayaro fever virus (MAYV) outbreak occurred in a settlement in Santa Barbara municipality, northern Brazil. Patients had rash, fever, and severe arthralgia lasting up to 7 days. Immunoglobulin M against MAYV was detected by ELISA in 36 persons; 3 MAYV isolates sequenced were characterized as genotype D.

Mayaro virus (MAYV) is a member of the family *Togaviridae* and the genus *Alphavirus*. Recent molecular studies have recognized 2 MAYV lineages: genotypes D and L (*1*). MAYV has been associated with a dengue-like illness with rash, fever, and severe arthralgia in tropical South America. Arthralgia lasts for several weeks and affects principally ankles, wrists, and toes, but also can affect major joints. MAYV causes a mild to moderately severe acute febrile illness of 3–5 days’ duration with uneventful recovery (*2*).

## The Study

In February 2008, an outbreak of a dengue-like illness was reported in the Pau D’arco settlement, 38 km from Belém, Para state, in the Brazilian Amazon (Appendix Figure). This rural community has 48 houses with ≈150 inhabitants, many of whom live in poor conditions. They reside in the middle of a native forest, in softwood houses, in the municipality of Santa Barbara (2007 population ≈14,439).

A total of 105 persons were examined in a house-to-house survey. They reported a febrile illness within the past 30 days, had a current febrile illness, or reported contact with persons with febrile illness. Fifty-three resided in the settlement (50 were agricultural workers), and 52 were agronomy students at a public university in Belém and had been training for a week at a field station adjacent to the settlement. The students slept in the station for a week; their activities included periodic visits to the settlement and sporadic ingression to the forest. Students and agricultural workers were bled weekly by convenience from March 17 through April 4, 2008. All serum samples were processed by ELISA for detection of immunoglobulin (Ig) M (*3*).

During the same diurnal period (9:00 am–3:00 pm), mosquitoes were captured in the settlement by using human bait on the ground and in the forest canopy (≈15 m high) near the residences. A total of 832 (49 lots) *Culicidae* mosquitoes were collected and frozen before being used for virus isolation. Of these, 188 (11 lots) were *Haemagogus janthinomys*, the main vector of MAYV; the remaining 644 (38 lots) were mainly members of the genera *Wyeomyia, Aedes, Sabethes*, and *Limatus*.

Newborn mice (*Mus musculus*) and C6/36 cells were inoculated with acute-phase serum from samples collected from febrile patients and pooled mosquitoes, as previously described (*4**,**5*). The inoculated animals and cells were observed daily, and the presence of virus was confirmed by complement fixation and immunofluorescent assays (*4*). Three MAYV strains were isolated: 2 from febrile persons and 1 from a pool with 2 *H. janthinomys* mosquitoes collected at ground level. All 3 strains were isolated with both assays.

**Figure 1:**
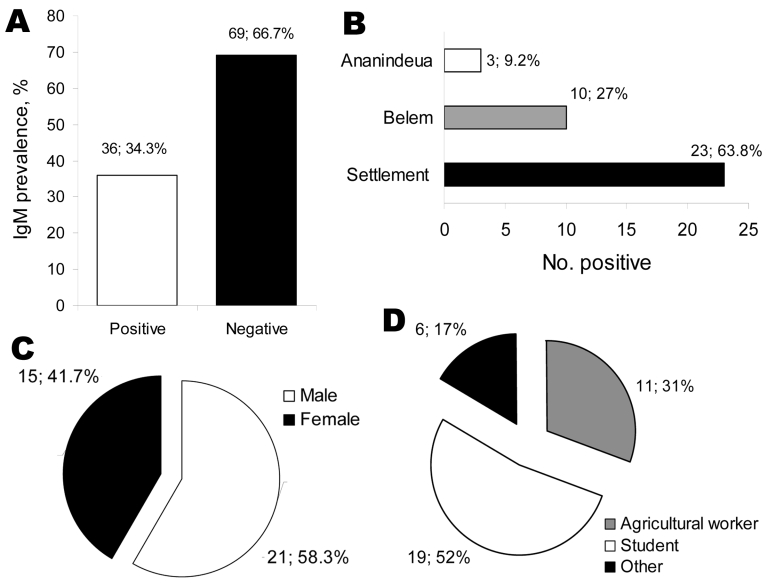
A) Prevalence of immunoglobulin (Ig) M against Mayaro virus in the studied population. B) Prevalence of IgM against Marayo virus according to area of residence. C) Patient sex. D) Patient work activities.

**Figure 2:**
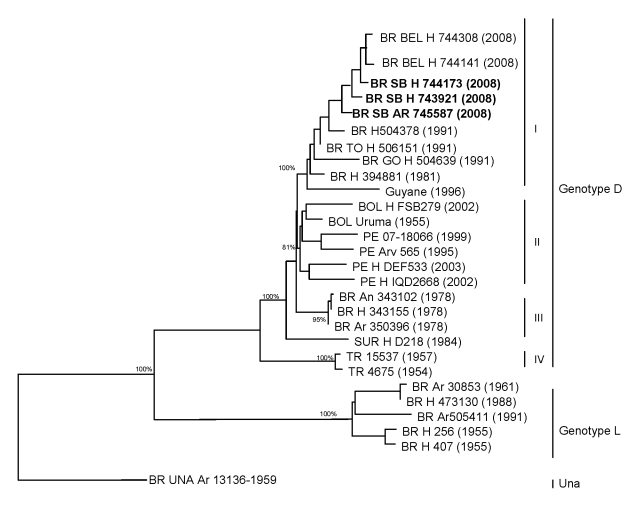
Comparison of genetic relationships among the Marayo virus strains sequenced in this study with those isolated in different areas of South America, periods of time, and hosts. Numbers above and within parentheses correspond to bootstrap support values for the specific clades. The Una virus was used as an outgroup to root the tree. BR, Brazil (BEL, Belém; SB, Santa Barbara [bold]; TO, Tocantins state); BOL, Bolivia; PE, Peru; SUR, Suriname; H, human; Ar, arthropod. Numbers in parentheses correspond to the year of isolation of each strain. Items in **boldface** indicate strains isolated in this study.

IgM was detected in 36 (34%) serum samples (Figure 1, panel A). Of those 36 samples, 23 (64%) were collected from residents of the settlement, and 13 (36%) were from residents of Belém and Ananindeua municipalities; these persons had visited the settlement area for a week (Figure 2, panel B). Persons with Mayaro fever ranged in age from 4 to 55 years, and 21 (58%) were male (Figure 1, panel C). Fifty-two percent of MAYV-positive persons were students, 31% were agriculturists, and 17% participated in other activities (Figure 1, panel D).

Of the 36 MAYV-infected persons, 33 were symptomatic. Illness was characterized by sudden onset of fever (100% of patients), arthralgia (89%), myalgia (75%), headache (64%), articular edema (58%), rash (49%), and retroocular pain (44%). Other less frequent symptoms were itching (33%), dizziness (25%), anorexia (22%), swollen lymph nodes (17%), and vomiting (4%).

Other common exanthematic illnesses in Brazil included in the differential diagnoses were dengue fever, rubella, B19 parvovirus, human herpesvirus 6, infectious mononucleosis, malaria, and yellow fever. Serologic results excluded these illnesses.

RNA was extracted by using the TRIZOL LS (Invitogen, Carlsbad, CA, USA) reagent method according to the manufacturer’s instructions. Envelope (E)2 and E1 genes of the MAYV genome were amplified by using a standard 1-step reverse transcription–PCR protocol, as previously described (*1*). The cDNA products were directly sequenced (*6*).

We conducted phylogenetic analysis by using the maximum parsimony (heuristic algorithm), neighbor-joining (Kimura 3-parameter and F84 corrections), and maximum-likelihood methods (*7*) implemented in the PAUP software (*8*) for the nucleotide sequences obtained for the isolates and representative members of other Mayaro-related viruses belonging to the genus *Alphavirus* available at GenBank (www.ncbi.nlm.nih.gov). Bootstrap resample method (1,000 replicates) and outgoup definition were used to provide confidence for the phylogenetic groups (*9*).

The 3 MAYV isolates were successfully sequenced, and the nucleotide sequences covering the 3′ E1 region, the entire E2 gene, and 3′ noncoding region (≈2,000 nt) were phylogenetically compared with other MAYV and Mayaro-related viruses isolated during different periods (1954–2008) and from different hosts (human and arthropods) in Brazil, Peru, French Guiana, Trinidad and Tobago, Suriname, and Bolivia (Figure 2).

The phylogram depicted a clear segregation of MAYV strains into 2 major groups: genotypes D and L (*1*). The strains isolated in Santa Barbara municipality were grouped together in genotype D within clade I. Genetically, these strains were closely related to a 1991 isolate from Tocantins state in northern Brazil. The strains isolated in Santa Barbara were similar to those isolated in Belém during the same period. Interestingly, the Santa Barbara and Belém strains differed from the Brazilian and prototype strains isolated in 1955 (Figure 2).

## Conclusions

MAYV has been isolated only in northern South America. Probably because of the short viremic period, it is sporadically isolated only during enzootic periods. However, during epidemics or epizootics, the number of isolates increase sharply (*10**,**11*). The few isolates obtained are intriguing and contrast with the high prevalence of specific antibodies in Pan-Amazonia; previous studies have shown widespread immunity in the Amazon, ranging from 5% to 60%. Positivity increases with age and is higher in rural and neighboring communities, as observed for the Amerindians (*2**,**12**,**13*).

In a previous outbreak in Belterra, several patients were too ill to continue their daily activities while febrile, and some even became prostrate. Moreover, these patients frequently reported severe arthralgia that led to temporary incapacitation (*13**,**14*).

Our data confirmed the occurrence of a Mayaro fever outbreak in the Pau D’Arco settlement. Clinically, the disease was similar to other outbreaks and characterized mainly by fever, arthralgia, myalgia, headache, rash, and dizziness (*2**,**13**–**15*). This outbreak was reported 17 years after the last episode of the disease described in the municipality of Benevides, which is closer (≈10 km) to Santa Barbara (P.F.C. Vasconcelos, unpub. data). The clinical and laboratory data from this MAYV outbreak caused by genotype D confirmed in Santa Barbara provide a better understanding of the MAYV molecular epidemiology in the Brazilian Amazon region.

## Supplementary Material

Appendix Figure A) Location of Pará State in northern Brazil; B) location of Belém region within Pará State; C) locations of 1) Santa Barbara and 2) Pau D'Arco settlements. PA-391, highway access to the municipality. Digital imaging was accessed in February 2008 at www.google.com.br/mapas.
