# Supplementary figures and images for: Mayaro Fever Virus, Brazilian Amazon
Source: Emerg Infect Dis. 2009 Nov;15(11):1830–2. doi: 10.3201/eid1511.090461 (PMC2857233; doi:10.3201/eid1511.090461)

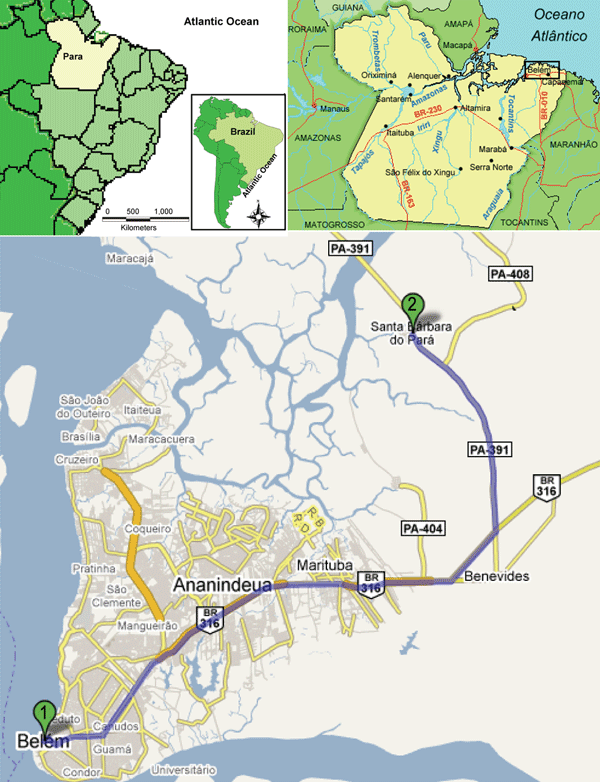

Supplement: Appendix Figure — A) Location of Pará State in northern Brazil; B) location of Belém region within Pará State; C) locations of 1) Santa Barbara and 2) Pau D'Arco settlements. PA-391, highway access to the municipality. Digital imaging was accessed in February 2008 at www.google.com.br/mapas. [file 09-0461_appF-s1.gif]
